# IL-8 correlates with reduced baseline femoral neck bone mineral density in adults with cystic fibrosis: a single center retrospective study

**DOI:** 10.1038/s41598-021-94883-1

**Published:** 2021-07-28

**Authors:** Grace Y. Lam, Sameer Desai, Joey Fu, Xun Yang Hu, Jiah Jang, Azita Goshtasebi, Shirin Kalyan, Bradley S. Quon

**Affiliations:** 1 Division of Pulmonary Medicine, University of Alberta, Edmonton, AB Canada; 2 School of Population and Public Health, University of British Columbia, Vancouver, BC Canada; 3 Division of Respiratory Medicine, St. Paul’s Hospital, Vancouver, BC Canada; 4 Centre for Heart Lung Innovation, St. Paul’s Hospital, University of British Columbia, 1081 Burrard Street, Vancouver, BC V6Z 1Y6 Canada; 5 Centre for Menstrual Cycle and Ovulation Research (CeMCOR), University of British Columbia, Vancouver, BC Canada; 6 Division of Endocrinology and Metabolism, Department of Medicine, University of British Columbia, Vancouver, BC Canada

**Keywords:** Cytokines, Interferons, Interleukins, Tumour-necrosis factors, Metabolic bone disease

## Abstract

Cystic fibrosis (CF) is a multi-system disease that is characterized by lung disease due to recurrent airway infection and inflammation. Endocrine complications, such as CF bone disease (CFBD), are increasingly identified as patients are living longer. The cause of CFBD is multifactorial with chronic systemic inflammation theorized to be a contributing factor. Thus, we attempted to identify inflammatory biomarkers that are associated with CFBD. We conducted a retrospective observational study of 56 adult patients with CF with an average percentage predictive forced expiratory volume in one second (ppFEV_1_) of 73.7% (standard deviation: 30.0) who underwent baseline serum analysis for osteoprotegerin (OPG) and pro-inflammatory biomarkers (IL-1β, IL-6, IL-8 and TNF-α), and had repeated dual-energy x-ray absorptiometry (DXA) scans separated by at least 2 years to examine correlations between serum biomarkers and bone mineral density (BMD) measurements. Univariate linear regression model analysis demonstrated that serum IL-1β and IL-8, but not other pro-inflammatory markers, were negatively correlated with baseline BMD results. However, after accounting for confounding variables, only the relationship between IL-8 and left femoral neck BMD remained statistically significant. Additionally, IL-8 level was associated with BMD decline over time. These results suggest that IL-8 might play a unique role in the pathophysiology of CFBD relative to other pro-inflammatory cytokines but further study is warranted before firm conclusions can be made.

## Introduction

Cystic Fibrosis (CF) is a multisystem disorder that manifests predominantly in the lungs and pancreas, but also in other organs such as the intestines and liver. CF is caused by mutations in the CF transmembrane conductance regulator (CFTR) protein, a channel that is responsible for transcellular movement of chloride and bicarbonate, regulating epithelial surface fluid and mucous viscosity^1^.

As the life expectancy of CF patients increases due to advances in treatment and management of their condition, other complications, such as osteoporosis and osteopenia, collectively referred to as CF-related bone disease (CFBD), are becoming more prevalent. Based on retrospective analyses, 24% of CF adult patients have CFBD^2,3^, which further increases to 55–65% in CF patients > 45 years of age^4,5^. In addition to age, other risk factors have been associated with low bone mineral density (BMD) including male sex, low body mass index (BMI), low lung function^6^, and having dysglycemia as a comorbidity^7^. Consequently, the CF Foundation recommends routine screening with the dual-energy X-ray absorptiometry (DXA) scan to assess for bone mineral density (BMD) starting from the age of 9^8^. Physiologically, bone density is a reflection of the mineral content in a given volume of bone, which is subject to a balance between osteoblasts mediating bone building activity and osteoclasts contributing to bone breakdown or resorption. There are likely multiple factors in CF that confers an imbalance between osteoblast and osteoclast activity that may result in CFBD, including CFTR dysfunction directly impairing new bone formation^9^ via reduced osteoblast activity^10^, as well as secondary causes, including malabsorption of calcium and vitamin D as a result of pancreatic insufficiency and recurrent pulmonary infections that lead to increased inflammatory bone resorption^2,4,11,12^.

The mechanism by which inflammation mediates bone resorption is complex and this relationship has been consistently documented in CF. At baseline, markers of bone mass turnover and formation, such as osteocalcin, are reduced in the CF population compared to the non-CF population^13–15^ while the receptor activator of nuclear factor kappa-Β ligand (RANK-L), a marker of bone resorption, is elevated in the CF population at baseline when compared to the non-CF population^13,16,17^. During periods of heightened systemic inflammation seen with pulmonary exacerbations, up-regulation of RANK-L and osteoclast activity have been demonstrated^13,16,17^. Circulating pro-inflammatory cytokines, such as interleukin (IL)-6, C-reactive protein (CRP), tumour necrosis factor (TNF)-α and IL-1β have been shown to be correlated with markers of bone resorption^11,12^ and inversely correlated to markers of bone formation^18^. Similarly, when human CF osteoblasts were provided with a pro-inflammatory stimulus, RANK-L production increased markedly while osteoprotegerin (OPG), a competitive inhibitor of RANK-L signalling, was found in relatively lower concentrations than RANK-L and as a result, osteoclastogenesis was favoured^16^. It remains unclear how these in vitro findings may impact clinical changes to BMD and CFBD development.

Here, we postulate that pro-inflammatory biomarkers, such as IL-1β, IL-6, IL-8, and TNF- α are inversely correlated to BMD measurements, the clinical manifestation of increased osteoclast activity. By longitudinally assessing a retrospective adult CF cohort with repeated DXA measurements over years, we further hypothesize that these pro-inflammatory markers may be associated with bone loss over time.

## Results

### Cohort selection

Based on the inclusion/exclusion criteria, 56 patients were included in this study. Of these, 5 subsequently went on to receive a lung transplant following baseline assessment. As BMD measurements after transplantation were excluded, 4 of these 5 individuals only contributed baseline BMD results to the analysis. Of the 52 patients who contributed repeated BMD measurements, the median number of BMD measurements per patient was 2 (minimum 2; maximum 5 measurements including baseline). The median time between first and last BMD measurements was 3.44 years (minimum 2 years and maximum 7.62 years).

### Clinical and biochemical assessments

#### Baseline clinical and biomarker characteristics

For the 56 patients included, 37 (66%) subjects were male, the mean age was 35.6 years (standard deviation [SD] 12.2), and ppFEV_1_ was 73.7% (SD 30.0) (Table 1). Twenty-one (38%) subjects were homozygous for ΔF508 and 19 (34%) were heterozygous for ΔF508. Forty-two (75%) subjects had pancreatic insufficiency, 14 (26%) had CF-related diabetes (CFRD), and 35 (63%) were sputum positive for *Pseudomonas* *aeruginosa* (PsA). Six (11%) patients were on a bisphosphonate at baseline. 6 patients were started on CFTR modulator therapy during the period of study where only 1 patient was on treatment at the time of their baseline BMD assessment. Baseline BMD measurements as well as serum OPG and pro-inflammatory biomarker levels are reported in Table 1.

**Table 1:** Overall baseline characteristics of study sample.

	N = 56
Age, mean (SD)	35.6 (12.2)
Female sex, N %)	19 (33.9%)
BMI, kg/m^2^ (SD)	23.1 (4.04)
**Genotype,** **N** **(%)**	
Heterozygous ΔF508	19 (33.9%)
Homozygous ΔF508	21 (37.5%)
Other	16 (28.6%)
Pancreatic insufficiency, N (%)^a^	42 (76.4%)
CFRD, N (%)^a^	14 (25.5%)
ppFEV_1_, mean (SD)	73.7 (30.1)
PsA growth in sputum, N (%)^a^	35 (63.6%)
Systemic corticosteroid use, N (%)^a^	1 (2%)
Osteopenic, N (%)^a^	19 (34.5%)
Osteoporotic, N (%)^a^	12 (21.8%)
Bisphosphonate use, N (%)^a^	5 (9.1%)
**BMD** **measurements**	
Right hip z score, g/cm^2^ (SD)	− 0.32 (1.03)
Left hip z score, g/cm^2^ (SD)	− 0.35 (1.04)
Total hip z score, g/cm^2^ (SD)	− 0.36 (0.92)
Right femoral neck z score, g/cm^2^ (SD)	− 0.58 (1.03)
Left femoral neck z score, g/cm^2^ (SD)	− 0.55 (1.08)
L1–L4 spine z score, g/cm^2^ (SD)	− 0.84 (1.38)
**Serum** **biomarkers**	
OPG, pg/ml (SD)	79.5 (31.4)
IL-1β, pg/ml (SD)	0.23 (0.18)
IL-6, pg/ml (SD)	2.58 (6.72)
IL-8, pg/ml (SD)	12.5 (40.3)
TNF-α, pg/ml (SD)	2.55 (1.18)

*BMI* body mass index, *CFRD* cystic fibrosis related diabetes, *ppFEV*_*1*_ percent predicted forced expiratory volume in one second, *PsA* *Pseudomonas* *aeruginosa*, *BMD* bone mineral density, *OPG* osteoprotegerin, *TNF-α* tumour necrosis factor alpha, *SD* standard deviation.

^a^One patient record was missing for these variables resulting in N = 55.

#### DXA measurements

At baseline, 12 (22%) subjects had BMD scores within the osteoporotic range while 19 (35%) were within the osteopenic range (Table 1). The majority of baseline z scores fell within normal limits (i.e. greater than − 1) (Supplemental Table S1). One subject had a history of previous fracture at the start of the study period.

### Correlation between baseline clinical characteristics and biomarkers

Univariate linear regression analysis was performed to identify potential correlations between serum biomarkers and clinical characteristics, which may act as confounders in the relationship between serum biomarkers and BMD. ppFEV_1_ was inversely correlated with TNF-α, IL-6 and IL-1β while BMI was inversely correlated with OPG and IL-1β (Supplemental Table S2). CFRD status was positively associated with IL-8 such that those with CFRD had higher IL-8 levels. No other significant relationships between clinical characteristics and biomarker levels were found.

### Correlation between baseline BMD measurements with baseline clinical characteristics and biomarkers

Univariate and multivariate linear regression modeling was next undertaken to determine which clinical characteristics or serum biomarkers were associated with baseline BMD results (Table 2).

**Table 2:** Univariate linear regression model analysis of baseline clinical and biomarker levels vs. baseline BMD measurements (n = 56).

	L1–L4 spine z score beta (95% CI)	Right hip z score	Left hip z score	Right femoral neck z score	Left femoral neck z score
Age (per 5-year increase)	− 0.08 (− 0.23, 0.08)	− 0.11 (− 0.22, 0.00)	− 0.09 (− 0.21, 0.02)	**−** **0.14** **(−** **0.25,** **−** 0.03)*	**−** **0.14** **(−** **0.25,** 0.02)*
Male sex	**−** **1.3** **(−** **2.0,** **−** 0.56)*	− 0.52 (− 1.1, 0.05)	− 0.5 (− 1.1, 0.08)	− 0.42 (− 1.00, 0.16)	− 0.47 (− 1.1, 0.13)
Homozygous ΔF508	0.75 (− 0.13, 1.6)	0.35 (− 0.32, 1.0)	0.21 (− 0.47, 0.89)	0.39 (− 0.28, 1.1)	0.37 (− 0.33, 1.1)
PI	0.07 (− 0.82, 0.96)	− 0.13 (− 0.8, 0.54)	− 0.07 (− 0.74, 0.60)	− 0.18 (− 0.85, 0.48)	− 0.08 (− 0.77, 0.62)
CFRD	− 0.44 (− 1.3, 0.42)	− 0.39 (− 1.0, 0.25)	− 0.31 (− 0.96, 0.33)	− 0.52 (− 1.2, 0.11)	− 0.52 (− 1.2, 0.14)
ppFEV_1_ (per 5% increase)	**0.07** **(0.01,** 0.13)*	**0.09** **(0.04,** 0.13)*	**0.09** **(0.05,** 0.13)*	**0.08** **(0.04,** 0.12)*	**0.08** **(0.04,** 0.13)*
BMI (per 1U increase)	**0.14** **(0.05,** 0.22)*	**0.13** **(0.07,** 0.19)*	**0.15** **(0.09,** 0.20)*	**0.12** **(0.06,** 0.18)*	**0.12** **(0.05,** 0.18)*
PsA growth	− 0.46 (− 1.2, 0.32)	− 0.46 (− 1.0, 0.11)	− 0.35 (− 0.93, 0.24)	**−** **0.59** **(−** **1.2,** **−** **0.02)**	**−** 0.57 (− 1.2, 0.03)
Log_10_ OPG	− 0.79 (− 3.3, 1.7)	− 0.59 (− 2.5, 1.3)	− 1.2 (− 3.1, 0.64)	− 0.80 (− 2.7, 1.1)	− 0.63 (− 2.6, 1.3)
Log_10_ IL-1β	− 1.2 (− 2.5, 0.12)	**−** **1.6** **(−** **2.5,** **−** 0.73)*	**−** **1.6** **(−** **2.6,** **−** 0.72)*	**−** **1.2** **(−** **2.2,** **−** 0.24)*	**−** **1.4** **(−** **2.4,** **−** 0.46)*
Log_10_ IL-6	0.28 (− 0.59, 1.1)	− 0.46 (− 1.1, 0.19)	− 0.48 (1.1, 0.16)	− 0.24 (− 0.89,0.41)	− 0.31 (− 0.99, 0.36)
Log_10_ IL-8	**−** **1.3** **(−** **2.4,** **−** 0.19)*	− **0.99** **(−** **1.8,** **−** 0.19)*	**−** **0.92** **(−** **1.7,** **−** 0.10)*	**−** **1.0** **(−** **1.8,** **−** 0.22)*	**−** **1.2** **(−** **2.0,** **−** 0.32)*
Log_10_ TNF-α	0.45 (− 1.8, 2.7)	− 0.71 (− 2.4, 0.98)	− 0.31 (− 2.0, 1.4)	− 0.59 (− 2.3, 1.1)	− 0.43 (− 2.2, 1.3)

Beta coefficient with standard error in parentheses.

*BMI* body mass index, *PI* pancreatic insufficiency, *CFRD* cystic fibrosis related diabetes, *ppFEV*_*1*_ percent predicted forced expiratory volume in one second, *PsA* *Pseudomonas* *aeruginosa*, *OPG* osteoprotegerin, *TNF-α* tumour necrosis factor alpha.

*P < 0.05.

In univariate analysis, BMI and ppFEV_1_ were positively associated with all baseline BMD measurements. Age was negatively associated with bilateral femoral neck BMD and male sex was negatively associated with L-spine BMD. Additionally, sputum positivity for *Pseudomonas* *aeruginosa* (PsA) was negatively associated with right femoral neck BMD. In multivariate analysis adjusted for other clinical covariates, age and male sex were negatively correlated with BMD and BMI remained positively correlated with BMD. Conversely, ppFEV_1_ no longer demonstrated a correlation with BMD following adjustment.

In univariate analysis, statistically significant negative correlations were observed between IL-8 with all BMD measurements and IL-1β with bilateral hip and femoral neck z scores (Table 2). In multivariate analysis adjusted for age, sex, ppFEV_1_, BMI and CFRD, both of these biomarkers demonstrated a weaker relationship with baseline BMD and only the negative correlation between IL-8 and left femoral neck z score remained statistically significant (Table 3).

**Table 3:** Multivariable linear regression models analysis of baseline biomarker features against baseline bone mineral density measures, adjusted for age, sex, baseline ppFEV_1_, BMI, and CFRD (n = 56).

Baseline measure	L1–L4 spine z score estimate (95% CI)	Right hip z score estimate (95% CI)	Left hip z score estimate (95% CI)	Right femoral neck z score estimate (95% CI)	Left femoral neck z score estimate (95% CI)
Age (per 5-year increase)^a^	− 0.15 (− 0.30 to 0.00)	**−** **0.16** **(−** **0.26** **to** **−** 0.05)*	**−** **0.15** **(−** **0.05−** **to** 0.01)*	**−** **0.19** **(−** **0.30−** **to** 0.08)*	**−** **0.18** **(−** **0.30−** **to** 0.06)*
Male sex^a^	**−** **1.3** **(−** **1.9** **to** **−** 0.66)*	**−** **0.54** **(−** **0.98** **to** **−** 0.11)*	**−** **0.51** **(−** **0.94** **to** **−** 0.08)*	− 0.43 (− 0.88 to 0.02)	− 0.48 (− 0.97 to 0.01)
ppFEV1 (per 5% increase)^a^	− 0.01 (− 0.08 to 0.06)	0.02 (− 0.03 to 0.07)	0.02 (− 0.02 to 0.07)	0.01 (− 0.04 to 0.06)	0.02 (− 0.03 to 0.07)
BMI (per 1U increase)^a^	**0.17** **(0.08** **to** 0.26)*	**0.15** **(0.08** **to** 0.21)*	**0.16** **(0.10** **to** 0.22)*	**0.14** **(0.08** **to** 0.21)*	**0.14** **(0.06** **to** 0.21)*
CFRD^a^	− 0.32 (− 1.0 to 0.4)	− 0.13 (− 0.63− 0.36)	− 0.06 (− 0.55 to 0.42)	− 0.25 (− 0.76 to 0.26)	− 0.24 (− 0.80 to 0.31)
Log_10_ OPG	− 0.75 (− 3.1 to 1.6)	0.50 (− 1.1 to 2.1)	− 0.08 (− 1.7 to 1.5)	0.16 (− 1.5 to 1.8)	0.29 (− 1.5 to 2.1)
Log_10_ IL-1β	− 0.24 (− 1.6 to 1.1)	− 0.85 (− 1.7 to 0.03)	− 0.71 (− 1.6 to 0.17)	− 0.43 (− 1.4 to 0.49)	− 0.78 (− 1.8 to 0.22)
Log_10_ IL-6	0.38 (− 0.45 to 1.2)	− 0.34 (− 0.90 to 0.23)	− 0.31 (− 0.87 to 0.25)	− 0.12 (− 0.71 to 0.47)	− 0.19 (− 0.83 to 0.45)
Log_10_ IL-8	− 0.68 (− 1.7 to 0.32)	− 0.58 (− 1.3 to 0.10)	− 0.50 (− 1.2 to 0.17)	− 0.64 (− 1.3 to 0.05)	**−** **0.78** **(−** **1.5** **to** **−** 0.04)*
Log_10_ TNF-α	0.64 (− 1.4 to 2.6)	− 0.25 (− 1.6 to 1.1)	0.22 (− 1.1 to 1.6)	0.02 (− 1.4 to 1.4)	0.19 (− 1.3 to 1.7)

*CI* confidence interval, *BMI* body mass index, *CFRD* cystic fibrosis related diabetes, *ppFEV*_*1*_ percent predicted forced expiratory volume in one second, *PsA* *Pseudomonas* *aeruginosa.*

*P < 0.05.

^a^Adjusted for other clinical covariates.

### Correlation between blood biomarkers and rate of BMD change over time

**Figure 1:**
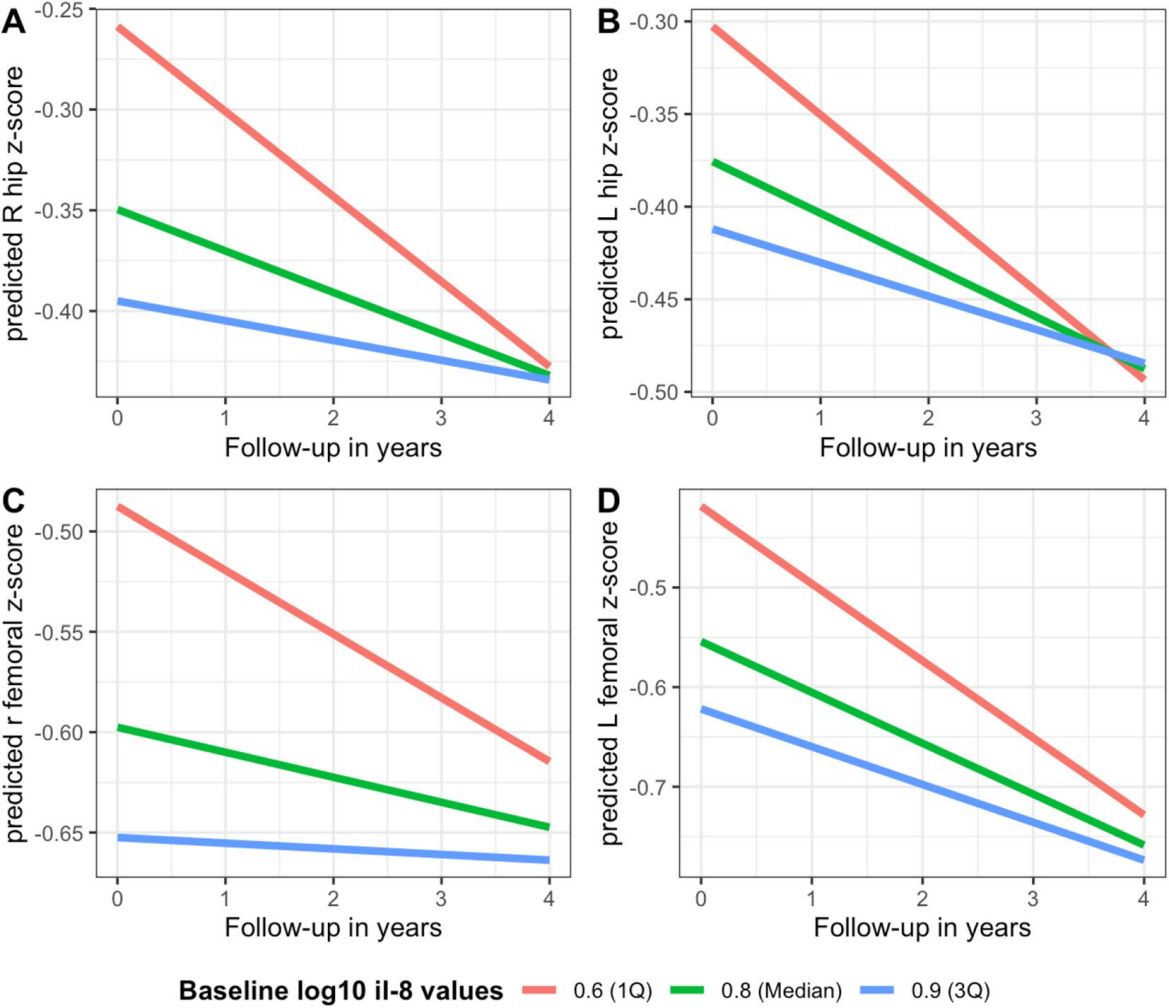
Predicted BMD measurements over time from linear mixed effect models using the first quartile, median, and the third quartile values of IL-8. (**A**) Right hip Z score; (**B**) left hip Z score; (**C**) right femoral neck Z score; (**D**) left femoral neck Z score. All other variables in the model were held constant at their means for continuous covariates, and categorical covariates at their proportions.

We next examined the relationship between clinical characteristics or baseline blood biomarkers with bone density change over time using a multivariate linear mixed effect models, adjusted for age, sex, ppFEV_1_, BMI and CFRD (Table 4). CFRD was positively associated with changes in L-spine and right hip BMD such that individuals with CFRD had an increase in BMD over time. As well, a positive correlation was found between IL-8 levels and changes in bilateral hip and femoral neck BMD. Categorical analysis of IL-8 levels divided into quartiles found that those with the highest IL-8 levels had the lowest BMD at baseline and the slowest rate of bone loss while those with the lowest IL-8 levels had the highest BMD at baseline and the fastest rate of bone loss over time (Fig. 1). Additionally, TNF-α was also positively correlated with changes in left femoral neck BMD over time (Table 4). No other relationships between clinical characteristics or baseline biomarkers and changes in BMD were demonstrated.

**Table 4:** Multivariate linear mixed effects model analysis of baseline blood biomarker levels vs. change in BMD measurements.

Baseline measures	L1–L4 spine z score estimate (95% CI)	Right hip z score estimate (95% CI)	Left hip z score estimate (95% CI)	Right femoral neck z score estimate (95% CI)	Left femoral neck z score estimate (95% CI)
Age (per 5-year increase)^a^	0.00 (− 0.00 to 0.00)	− 0.00 (− 0.01 to 0.01)	− 0.00 (0.01 to 0.01)	− 0.00 (− 0.01 to 0.01)	− 0.00 (− 0.02 to 0.01)
Male sex^a^	0.07 (− 0.01 to 0.15)	0.04 (− 0.02 to 0.10)	0.05 (− 0.01 to 0.10)	0.05 (− 0.02 to 0.11)	0.05 (− 0.01 to 0.11)
ppFEV_1_ (per 5% increase)^a^	0.00 (− 0.00 to 0.01)	0.00 (− 0.00 to 0.01)	0.00 (− 0.00 to 0.01)	− 0.00 (− 0.01 to 0.00)	− 0.00 (− 0.01 to 0.00)
BMI (per 1U increase)^a^	0.00 (− 0.01 to 0.01)	− 0.00 (− 0.01 to 0.00)	− 0.00 (− 0.01 to 0.01)	− 0.00 (− 0.01 to 0.01)	0.00 (− 0.00 to 0.01)
CFRD^a^	**0.12** **(0.04** **to** 0.20)*	**0.08** **(0.02** **to** 0.13)*	0.04 (− 0.01 to 0.10)	0.06 (− 0.00 to 0.13)	0.05 (− 0.02 to 0.11)
Log_10_ OPG	0.06 (− 0.22 to 0.34)	0.12 (− 0.06 to 0.31)	0.11 (− 0.08 to 0.30)	0.12 (− 0.10 to 0.34)	0.02 (− 0.20 to 0.23)
Log_10_ IL-1β	0.02 (− 0.10 to 0.14)	0.07 (− 0.01 to 0.16)	0.01 (− 0.07 to 0.09)	0.08 (− 0.02 to 0.17)	0.07 (− 0.02 to 0.16)
Log_10_ IL-6	− 0.04 (− 0.11 to 0.04)	0.01 (− 0.04 to 0.06)	0.00 (− 0.05 to 0.05)	0.00 (− 0.06 to 0.06)	0.03 (− 0.03 to 0.08)
Log_10_ IL-8	0.09 (− 0.00 to 0.17)	**0.11** **(0.05** **to** 0.16)*	**0.10** **(0.04** **to** 0.15)*	**0.10** **(0.03** **to** 0.16)*	**0.13** **(0.07** **to** 0.19)*
Log_10_ TNF-α	0.04 (− 0.24 to 0.33)	0.12 (− 0.07 to 0.31)	0.12 (− 0.07 to 0.31)	0.17 (− 0.05 to 0.39)	**0.25** **(0.04** **to** 0.46)*

Estimates represent change in BMD per year adjusted for age, sex, baseline ppFEV_1_, BMI, and CFRD.

*CI* confidence interval, *BMI* body mass index, *CFRD* cystic fibrosis related diabetes, *ppFEV*_*1*_ percent predicted forced expiratory volume in one second, *PsA* *Pseudomonas* *aeruginosa.*

*P < 0.05.

^a^Adjusted for other clinical covariates.

Six subjects were on bisphosphonates at baseline, which could have attenuated the rate of BMD change. Similarly, six individuals were initiated on CFTR modulators at various points during this study with one individual already on treatment at baseline, which may also have affected the rate of BMD change. Therefore, sensitivity analyses were performed to determine if individuals on bisphosphonates or CFTR modulators affected the multivariate analysis findings (Supplemental Table S3, S4). Exclusion of these subjects resulted in no significant change and the positive associations remained between IL-8 and bilateral hip and femoral neck BMD changes.

## Discussion

CFBD is an increasingly common complication seen in CF patients caused in part by increased levels of systemic inflammation stemming from recurrent pulmonary exacerbations. While prior studies have demonstrated a relationship between systemic inflammation and markers of bone resorption during heightened states of inflammation (i.e. pulmonary exacerbations)^12^, in this study, we examined the association between systemic inflammation during clinical stability and BMD. Overall, our findings suggest that inflammation is associated with a reduced BMD at baseline but is not likely to be predictive of bone loss over time.

The pro-inflammatory cytokines examined in this study were selected due to their putative roles in the pathogenesis of inflammatory bone disease and osteoporosis by synergistically stimulating osteoclastogenesis and/or inhibiting osteoblasts^19^. Both serum IL-8 and IL-1β were found to be inversely correlated with baseline BMD in the crude analysis but only the relationship between IL-8 and left femoral neck BMD remained statistically significant following adjustment for clinical covariates. While it is possible that a larger sample size might have identified additional statistically significant relationships between inflammatory markers and baseline BMD, the relationship observed between serum IL-8 and left femoral neck BMD suggests that it may possibly play a unique role in the pathogenesis of CFBD. Based on the results of an in vitro study by Le Heron et al., inhibiting CFTR in osteoblasts results in the enhanced production of IL-8 and a reduction in OPG and thus may contribute to inflammation-driven bone loss^9^. This important finding suggests that CFTR dysfunction can directly impact on bone health, independent of inflammation stemming from pulmonary exacerbations, and that IL-8 could be one of the key mediators of this effect. Thus, the hypothesized role of IL-8 in the pathogenesis of CFBD deserves further study and validation.

Contrary to expectations based on our cross-sectional findings, the group of individuals with the highest IL-8 levels experienced a slower rate of bone loss over time relative to the group with the lowest IL-8 levels (Fig. 1). However, the group with the highest IL-8 levels started with a lower BMD to begin with and therefore the lower rate of change in BMD may have been a result of the “floor effect” as those individuals with the lowest BMD had the least to lose, whereas those individuals with the highest BMD had the most to lose. Furthermore, those with lowest baseline BMD may have received more aggressive interventions, such as use of bisphosphonates, over the course of the observational study compared to those with normal baseline BMD which could have attenuated the rate of bone loss in the group with the highest IL-8 level.

There are a number of limitations to our study that should be considered. As mentioned, this is a single center observational study focused on adults with a relatively small sample size and therefore it might be limited in terms of generalizability. In addition, BMD measurements were performed at different sites on different DEXA scanners throughout the study and therefore there may have been analytical differences in the reported BMD measurements. However, since the clinical variables that we identified to be correlated with BMD is consistent with what has been reported in the literature, the use of different DEXA scanners likely did not introduce significant variability to the data. Finally, our study focused on baseline biomarker measurements and thus it is not clear how ongoing fluctuations in these pro-inflammatory cytokines, such as during pulmonary exacerbations, might alter BMD over time and therefore a longitudinal study is required to examine this further.

In conclusion, serum IL-8 correlates with femoral bone BMD at baseline and may play an important role in inflammation-related bone disease in CF but further study is required before firm conclusions can be made. Given that both CFTR dysfunction and pulmonary exacerbations can result in an elevation of IL-8^9^, CFTR modulators may have a beneficial effect on bone health by virtue of improving CFTR function and reducing pulmonary exacerbations. Future studies are required to fully understand the impact of CFTR modulators on CFBD.

## Methods

### Patient inclusion and exclusion criteria

Patients from the St. Paul’s Hospital Adult CF Clinic (Vancouver, Canada) enrolled in a single-centre CF Biomarker study between 2012 and 2018 had serum samples collected during non-consecutive stable clinic visits. The inclusion criteria for this sub-study focused on bone health were: patients aged 19 years or older with a baseline DXA scan performed within one year of a stable visit blood sample and at least one follow-up DXA scan at least 2 years later. Patients on immunosuppressants or with a history of lung transplantation at baseline were excluded. If a patient subsequently underwent lung transplantation after baseline, BMD measurements collected after transplantation were excluded. The research protocol was approved by the University of British Columbia Providence Health Care Research Institute Research Ethics Board (REB#H16-01063). Informed patient consent was obtained for the use of blood samples from the CF Biomarker study (REB#H12-00835). This project was conducted in full accordance with the above research ethics board guidelines and regulations and in accordance with the Declaration of Helsinki.

### Clinical data collection

Clinical characteristics corresponding to baseline blood sample collection were collected through chart review and included: patient age, genotype, pancreatic status, weight, height, disease co-morbidities, lung transplant status, medications, percentage predicted forced expiratory volume in one second (ppFEV_1_) and forced vital capacity in liters (FVC). DXA scan results measured as part of clinical care were collected from electronic medical records. Lumbar spine, bilateral hip, and bilateral femoral neck BMD were reported in g/cm^2^ and as z scores.

### Serum analysis

Blood samples stored at − 80 °C were thawed and underwent batched measurement. OPG, a marker of bone formation, and pro-inflammatory markers (IL-1β, IL-6, IL-8 and TNF-α) were analyzed. Serum OPG levels were measured using Human TNFRSF11B (OPG) ELISA Kits (Thermo Scientific, Fredrick, MD, USA) with an assay sensitivity of 1 pg/mL. Serum pro-inflammatory cytokine levels (IL-1β, IL-6, IL-8, and TNF-α) were measured using the V-Plex Human Proinflammatory Panel II (4-Plex) kits (Meso Scale Diagnostics, Rockville, ML, USA) with an assay sensitivity of 0.04–0.07 pg/mL. Assay mean coefficient of variability (CV) for OPG, TNF-α, IL-6, IL-8 and IL-1β were 6.15%, 8.31%, 11.00%, 12.60% and 5.45%, respectively.

### Statistical analysis

Clinical characteristics were described using simple descriptive statistics (e.g. means and percentages). A univariate linear regression model was used to estimate crude relationships between baseline blood biomarker levels or clinical characteristics and baseline BMD measurements. A multivariable linear regression model was used to determine relationships between baseline blood biomarkers levels or clinical characteristics and baseline BMD measurements after adjustment for covariates. Multivariate linear mixed effect regression models, which accounts for correlated BMD from each patient, was then used to estimate the correlation between each of the baseline biomarkers or clinical characteristics and rate of BMD change from baseline to the last BMD measured by introducing an interaction term (i.e., slope between repeated BMD measurements over time). Random intercepts for within-patient variation, comprised of an unstructured covariance pattern, was treated as a random effect in the mixed effects model and known or suspected predictors of bone loss (age, sex, BMI, ppFEV_1_, CFRD status) were also included^20,21^.

Serum biomarker levels were log_10_ transformed prior to analysis. The level of significance was set at *P* < 0.05 for all statistical analyses and all reported *P* values reflect two-tailed tests. All analyses were conducted using R version 3.5.2 statistical programming^22^ and “lme4” package^23^.

## Supplementary Information

Supplementary Information 1.
