# Supplementary material for: IL-8 correlates with reduced baseline femoral neck bone mineral density in adults with cystic fibrosis: a single center retrospective study
Source: Sci Rep. 2021 Jul 28;11:15405. doi: 10.1038/s41598-021-94883-1 (PMC8319414; doi:10.1038/s41598-021-94883-1)
Supplement: Supplementary file 1 — Supplementary Information 1. [file 41598_2021_94883_MOESM1_ESM.docx]

**Supplemental Table 1. Number of Patients with Baseline Hip, Femoral neck and L-spine z-scores > -1, Between -1 to -2 and < -2 (N = 56)**

|  | z-score > -1 | z-score -1 to -2 | z-score < -2 |
| --- | --- | --- | --- |
| Left hip | 39 (70%) | 14 (25%) | 3 (5%) |
| Total hip | 37 (71%) | 13 (25%) | 2 (4%) |
| Right femoral neck | 34 (61%) | 17 (30%) | 5 (9%) |
| Left femoral neck | 37 (66%) | 13 (23%) | 6 (11%) |
| L1-4 spine | 29 (52%) | 16 (29%) | 11 (20%) |

**Supplemental Table 2. Univariate Linear Regression Model Analysis** **of Baseline Clinical vs. Biomarker Levels (n=56)**

|  | Log_10_ OPG (95% CI) | Log_10_ IL-1β (95% CI) | Log_10_ IL-6 (95% CI) | Log_10_ IL-8 (95% CI) | Log_10_ TNF-α (95% CI) |
| --- | --- | --- | --- | --- | --- |
| Age (per 5-yr increase) | 0.00  (-0.01, 0.02) | -0.01 (-0.04, 0.02) | 0.01 (-0.04, 0.05) | -0.01  (-0.04, 0.03) | 0.01 (-0.01, 0.03) |
| Male Sex | -0.07 (-0.16, 0.01) | 0.06 (-0.10, 0.22) | -0.06 (-0.30, 0.19) | 0.12  (-0.07, 0.31) | -0.03 (-0.12, 0.07) |
| Homozygous ΔF508 | 0.07  (-0.02, 0.17) | -0.12  (-0.30, 0.06) | -0.15  (-0.42, 0.12) | -0.16  (-0.36, 0.04) | -0.06  (-0.16, 0.05) |
| PI | -0.03  (-0.12, 0.07) | 0.03  (-0.15, 0.21) | 0.14  (-0.14, 0.41) | 0.00  (-0.21, 0.22) | -0.05  (-0.16, 0.05) |
| CFRD | -0.01 (-0.11, 0.08) | -0.07 (-0.24, 0.11) | -0.07 (-0.34, 0.20) | **0.22 (0.02, 0.42)*** | 0.04 (-0.06, 0.14) |
| ppFEV_1_ (per 5% increase) | -0.01  (-0.01, 0.00) | **-0.02**  **(-0.03,-0.01)**  ******* | **-0.03**  **(-0.05,-0.01)**  ******* | -0.01 (-0.03, 0.00) | **-0.01**  **(-0.02, 0.00)*** |
| BMI (per 1U increase) | **-0.01 (-0.02, 0.00)*** | **-0.03**  **(-0.05,-0.01)**  ******* | -0.01  (-0.04, 0.02) | -0.01 (-0.04, 0.01) | 0.00  (-0.01, 0.01) |
| PsA growth | -0.04 (-0.12, 0.04) | 0.06 (-0.10, 0.21) | -0.06 (-0.30, 0.19) | 0.10  (-0.09, 0.29) | 0.05  (-0.04, 0.14) |

* *P* < 0.05, *** *P* < 0.001

Beta coefficient with 95% confidence interval in parentheses

BMI = body mass index; PI = pancreatic insufficiency; CFRD = Cystic fibrosis related diabetes; ppFEV_1_ percent predicted forced expiratory volume in one second; PsA = *Pseudomonas aeruginosa*; OPG = osteoprotegerin; TNF-α = Tumour necrosis factor alpha

**Supplemental Table 3. Sensitivity Analysis examining the Effect of excluding 6 patients on Bisphosphonates in the relationship between Baseline Blood Biomarker Levels vs. Change in BMD Measurements. Estimates represent change in BMD per year adjusted for age, sex, baseline** **ppFEV_1_, BMI, and CFRD**

|  | L1-L4 spine Z-score (95% CI) | Right hip z-score  (95% CI) | Left hip z-score  (95% CI) | Right femoral neck z-score  (95% CI) | Left femoral neck z-score  (95% CI) |
| --- | --- | --- | --- | --- | --- |
| Baseline Log_10_ OPG | 0.08  (-0.21-0.37) | 0.12  (-0.07-0.32) | 0.09  (-0.10-0.27) | 0.12  (-0.11-0.34) | 0.01  (-0.21-0.24) |
| Baseline Log_10_ IL-1β | 0.02  (-0.12-0.15) | 0.08  (-0.01-0.17) | 0.06  (-0.03-0.15) | 0.09  (-0.02-0.19) | 0.09  (-0.01-0.20) |
| Baseline Log_10_ IL-6 | -0.04  (-0.12-0.04) | 0.01  (-0.04-0.06) | 0.01  (-0.04-0.07) | -0.00  (-0.06-0.06) | 0.03  (-0.03-0.09) |
| Baseline Log_10_ IL-8 | 0.08  (-0.01-0.17) | **0.11**  **(0.05-0.17)**  ******* | **0.11**  **(0.06-0.16)**  ******* | **0.10**  **(0.03-0.17)**  ****** | **0.14**  **(0.07-0.20)**  ******* |
| Baseline Log_10_ TNF-α | 0.03  (-0.28-0.33) | 0.13  (-0.07-0.34) | 0.16  (-0.04-0.35) | 0.18  (-0.05-0.42) | **0.28**  **(0.05-0.50)**  ***** |

* *P* < 0.05; ** *P* <0.01; *** *P* < 0.001

CI = confidence interval; BMI = body mass index; CFRD = Cystic fibrosis related diabetes; ppFEV_1_ percent predicted forced expiratory volume in one second

**Supplemental Table 4. Sensitivity Analysis examining the Effect of excluding 6 patients on CFTR Modulators in the relationship between Baseline Blood Biomarker Levels vs. Change in BMD Measurements. Estimates represent change in BMD per year adjusted for age, sex, baseline** **ppFEV_1_, BMI, and CFRD**

|  | L1-L4 spine Z-score (95% CI) | Right hip z-score  (95% CI) | Left hip z-score  (95% CI) | Right femoral neck z-score  (95% CI) | Left femoral neck z-score  (95% CI) |
| --- | --- | --- | --- | --- | --- |
| Baseline Log_10_ OPG | 0.04  (-0.28-0.37) | 0.11  (-0.11-0.33) | 0.10  (-0.12-0.33) | 0.09  (-0.16-0.34) | 0.05  (-0.20-0.30) |
| Baseline Log_10_ IL-1β | 0.00  (-0.14-0.15) | 0.05  (-0.05-0.16) | -0.02  (-0.11-0.08) | 0.05  (-0.06-0.17) | 0.09  (-0.02-0.20) |
| Baseline Log_10_ IL-6 | -0.05  (-0.13-0.03) | 0.00  (-0.06-0.05) | -0.01  (-0.06-0.05) | -0.02  (-0.08-0.04) | 0.03  (-0.03-0.09) |
| Baseline Log_10_ IL-8 | 0.09  (0.00-0.18) | **0.11**  **(0.05-0.16) ***** | **0.10**  **(0.04-0.16) **** | **0.09**  **(0.03-0.16) **** | **0.13**  **(0.07-0.19) ***** |
| Baseline Log_10_ TNF-α | 0.04  (-0.28-0.36) | 0.13  (-0.08-0.35) | 0.13  (-0.09-0.35) | 0.18  (-0.06-0.42) | **0.26**  **(0.02-0.49)**  ***** |

* *P* < 0.05; ** *P* <0.01; *** *P* < 0.001

CI = confidence interval
